# Pembrolizumab-Induced Psoriasis in Metastatic Melanoma: Activity and Safety of Apremilast, a Case Report

**DOI:** 10.3389/fonc.2020.579445

**Published:** 2020-10-14

**Authors:** Maria Anna Siciliano, Stefano Dastoli, Maria d’Apolito, Nicoletta Staropoli, Pierfrancesco Tassone, Pierosandro Tagliaferri, Vito Barbieri

**Affiliations:** ^1^Department of Experimental and Clinical Medicine, Magna Graecia University, Catanzaro, Italy; ^2^Dermatology Unit, Mater Domini Hospital, Catanzaro, Italy; ^3^Department of Health Sciences, Magna Graecia University, Catanzaro, Italy; ^4^Medical Oncology Unit, Mater Domini Hospital, Catanzaro, Italy; ^5^Translational Medical Oncology Unit, Mater Domini Hospital, Catanzaro, Italy

**Keywords:** immune-related adverse events, cutaneous immune-related adverse events, psoriasis, checkpoint inhibitors, immunotherapy, Pembrolizumab, melanoma, Apremilast

## Abstract

**Background:**

Immune checkpoint inhibitors targeting cytotoxic T lymphocyte-associated antigen 4 (CTLA-4), programmed death-1 receptor (PD-1), and programmed death-1 receptor and its ligand (PD-L1) increased the survival of patients affected by metastatic malignant melanoma. Due to their mechanism of action, these drugs are associated with a unique toxicity profile. Indeed, immune-related adverse events (irAEs) present a wide clinical spectrum representing the Achilles’ heel of immunotherapy. Overall, cutaneous toxicities are among the most common irAEs. Immunomodulatory drugs are used for the management of irAEs and can theoretically lead to tumor escape.

**Case Presentation:**

We report the case of a 75-year-old man with metastatic melanoma receiving the anti-PD1 Pembrolizumab therapy. After 10 treatment cycles, the patient came to our clinic with itchy psoriatic manifestations widespread >30% of the body surface [12.3 Psoriasis Area and Severity Index (PASI) score] that negatively impacted on the patient’s quality of life and compliance with immunotherapy. Additionally, he had no positive personal history of psoriasis. Given the severity of the cutaneous manifestations, in a multidisciplinary approach, Apremilast (an oral small molecule PDE4 inhibitor) was started. Furthermore, Pembrolizumab was interrupted for 4 weeks until the improvement of skin lesions and the disappearance of itching. Immunosuppressive methylprednisolone therapy was initiated with a dose of 16 mg/die; then, this initial dose was progressively reduced until discontinuation. After 10 months, the patient had a good general clinical condition with psoriasis complete remission. Moreover, positron emission tomography (PET) and computed tomography (CT) scans showed complete response by immune Response Evaluation Criteria in Solid Tumors (iRECIST).

**Conclusion:**

To the best of our knowledge, this is the first report on the safety and efficacy of Apremilast for the treatment of immunotherapy-induced psoriasis in metastatic melanoma.

## Background

Immune checkpoint blockade targeting negative regulator proteins, such as cytotoxic T lymphocyte-associated antigen 4 (CTLA-4), programmed death-1 receptor (PD-1), and programmed death-1 receptor and its ligand (PD-L1), is presently the standard of care for an increasing number of malignancies. There is now increasing information on the mechanisms underlying the harnessing of the patient’s immune system and leading to immune escape.

Over the last decades, the treatment of metastatic malignant melanoma (MM) underwent dramatic enhancement with the approval of targeted therapy and immunotherapy. With a median follow-up of 55 months, in the analysis of Keynote-001, the estimated overall survival (OS) was 34% in all patients and 41% in treatment-naïve patients; median OS was 23.8 months (95% CI, 20.2–30.4) and 38.6 months (95% CI, 27.2–not reached), respectively (1). In the 5-year Checkmate-067 survival analysis, sustained long-term OS was observed in a greater percentage of patients who received nivolumab plus ipilimumab or nivolumab alone than those who received ipilimumab alone. At a minimum follow-up of 60 months, the median OS had not been reached in the group receiving combined treatment (2).

By increasing the activity of the immune system, checkpoint inhibitors can have inflammatory side effects, called immune-related adverse events (irAEs), presenting a wide clinical spectrum. A multidisciplinary approach is in most cases needed for the clinical management of irAEs (3).

We here report a clinical case of a patient with a metastatic melanoma who developed marked psoriasis following treatment with Pembrolizumab. After a multidisciplinary discussion, this patient has been treated with Apremilast.

## Case Presentation

**FIGURE 1:**
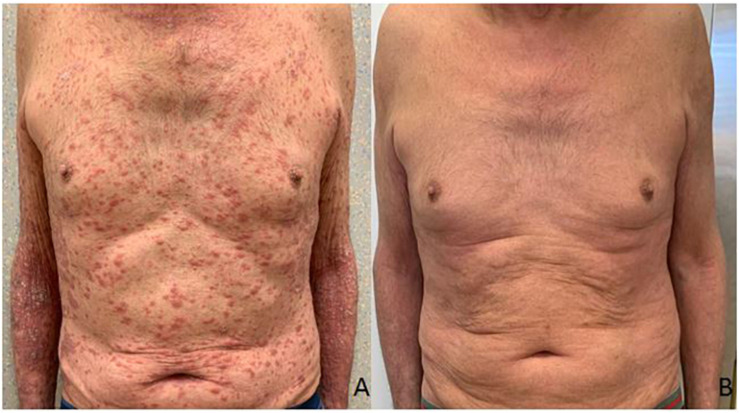
Clinical presentation of the patient before the treatment with Apremilast **(A)**. Resolution of psoriasis after the treatment with Apremilast **(B)**.

**FIGURE 2:**
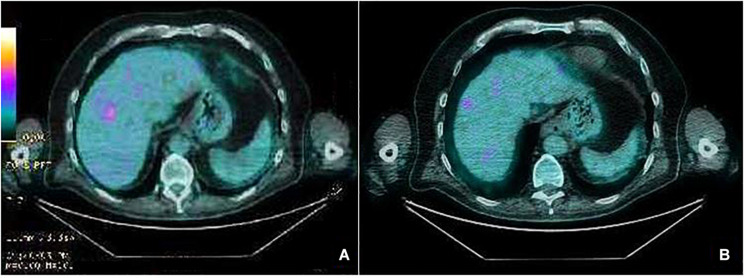
PET scan liver images before starting treatment with Pembrolizumab showing a pathologic standardized uptake value (SUV) **(A)**. After 18 cycles of treatment, PET scan showing iRECIST complete response **(B)**.

A 75-year-old man with a diagnosis of cutaneous nodular ulcerated melanoma of the left arm that measured 6.8 mm was referred to our oncology outpatient in October 2018. The tumor infiltrated the papillary/reticular dermal interface (Clark’s level III) and the side margins of the excision. The histological examination showed features of Breslow’s high-risk (thickness 2.7 mm) and the presence of inflammatory infiltrate. A positron emission tomography (PET) scan revealed pathological uptake of 18F-fluorodeoxyglucose (18-FDG) in the liver (Figure 2A). The mutational status of BRAF was wild type. The patient was treated with a flat dose of intravenous Pembrolizumab (200 mg every 3 weeks). Moreover, he had no history of autoimmune disease. After the 10th cycle, he developed psoriasis with itchy, multiple, well-marked, and raised, red plaque spread on >30% of the body surface [12.3 Psoriasis Area and Severity Index (PASI) score] (Figure 1A). This irAE had a significant negative impact on the patient’s quality of life and on his compliance with immunotherapy.

**FIGURE 3:**
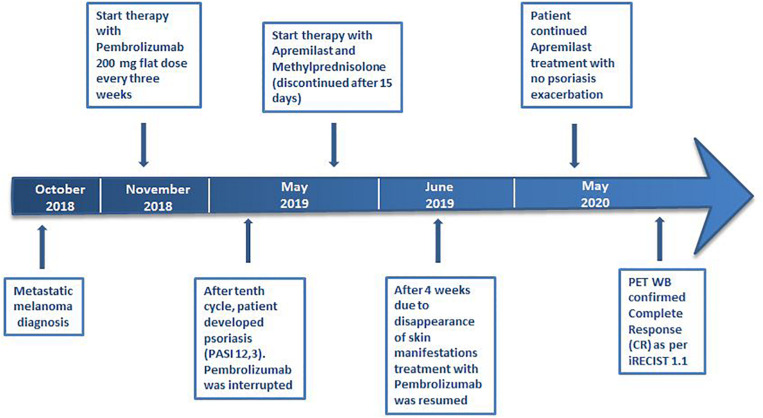
Case report timeline.

Due to the severity of psoriasis, following a multidisciplinary discussion of the clinical case, the patient underwent treatment with Apremilast at an initial 5-day titration dose of 10 mg and then at a maintenance dose of 30 mg twice daily. In order to obtain an immediate response, corticosteroid therapy was prescribed with immunosuppressive methylprednisolone at a dose of 16 mg/die for 5 days, followed by progressive dose reduction until discontinuation 15 days later. Furthermore, Pembrolizumab was interrupted for 4 weeks until the improvement of skin manifestations and disappearance of itching (Figure 1B). After 10 months of treatment with Apremilast and Pembrolizumab, the patient had a good clinical condition and acceptable quality of life, with no relapse of the psoriasis plaques. Moreover, PET and computed tomography (CT) scans showed no evidence of previously described melanoma lesions (complete response as per iRECIST) (Figure 2B). A chronological summary of the case report is shown in Figure 3.

## Discussion

Immunotherapy elicits the patient’s own immune system against cancer cells, reverting cancer-mediated immune suppression. Inhibitory synapses play a key role in adaptive immunity, self-tolerance, and prevention of autoimmunity.

Checkpoint inhibitors target regulatory receptors (CTLA-4, PD-1, or its ligand PD-L1, CD80, and CD86), inhibiting the preexisting immune response and enhancing antitumor immunity in several ways: T-cell activation and reverse of T-cell exhaustion and release and production of pro-inflammatory cytokines reprogramming both immune and non-immune cells in the tumor microenvironment.

Due to their mechanism of action, immune checkpoint inhibitors are associated with a unique toxicity profile representing the Achilles’ heel of these drugs (4, 5).

IrAEs management is extremely challenging because of the difficult equilibrium between active anti-cancer immunity and the risk of off-target autoimmunity due to a remarkable intersection between the involved effector pathways. Moreover, some immunomodulatory drugs used for the management of irAEs can theoretically lead to tumor escape (6, 7).

The mechanisms underlying the onset of irAEs are not completely understood. However, these are a breach of self-tolerance with the generation of autoreactive T- and B-cells escaped from central tolerance, cross-antigen reactivity, the release of pro-inflammatory cytokines/chemokines, off-target effects, and specific bacterial species in the microbiome (6).

Cutaneous toxicities are among the most common irAEs among all immune checkpoint inhibitors corresponding to a class effect. Since the beginning of immunotherapy, the time to onset of response varies between 2 weeks and several months. Cutaneous irAEs are more prevalent, severe, and earlier with combination strategies. Clinical presentations are broad-spectrum: itch, vitiligo, maculopapular rashes, bullous pemphigoid, and severe but rare cutaneous adverse reactions (Stevens-Johnson syndrome, toxic epidermal necrolysis, etc.) (8, 9).

Psoriasis is a chronic skin condition caused by a dysregulation of the immune system with a network of pro-inflammatory cytokines released by both immune cells (adaptive and innate) and non-immune cells. One of the secondary messengers implicated in intracellular signaling and negative regulation of inflammatory gene expression is the cyclic nucleotide cyclic adenosine monophosphate (cAMP). Intracellular levels of cAMP are tightly controlled by adenylyl cyclase, which promotes cAMP synthesis, and by cyclic nucleotide phosphodiesterases (PDEs), which hydrolyze cAMP. PDE-4 specifically hydrolyzes cAMP and represents the principal PDE in the inflammatory cells. Furthermore, PDE4 is highly expressed in the cell types involved in psoriasis, such as keratinocytes, vascular endothelium, and synovium (10). Apremilast (Otezla, Amgen) is a small oral molecule that selectively inhibits PDE-4. This enzyme hydrolyzes the cAMP increasing intracellular levels in immune cells, activates the protein kinase A (PKA), up-regulates the cAMP-response element (CRE)-containing genes, activates the transcription factor-1 (ATF-1), and inhibits the nuclear factor-kappa B (NF-kB) pathway. The result is the down-regulation of pro-inflammatory cytokines/chemokines, such as tumor necrosis factor (TNF)-a, interferon (IFN)-gamma, interleukin (IL)-23, IL-12, chemokine (C-X-C motif) ligand 9 (CXCL9), chemokine (C-X-C motif) ligand 10 (CXCL10), and carbon tetrachloride (C-C motif) ligand 4 (CCL4), and increase of anti-inflammatory cytokines, such as IL-10. These immune modulations result in reduced immune cell infiltration and changes in resident cells of the skin and joints. Moreover, in a transgenic murine model, Apremilast did not affect the clonal expansion of either T- or B-cells (11–13).

This oral small molecule has shown efficacy in psoriasis, psoriatic arthritis, and Behcet’s syndrome (14, 15). In March 2014, the Food and Drug Administration (FDA) approved this immunomodulatory drug for the treatment of adult patients with active psoriasis and other dermatological conditions, such as psoriatic arthritis refractory/intolerant to other local or systemic treatments (corticosteroids, methotrexate, cyclosporine, etc.). Apremilast is recommended at a dosage of 30 mg twice daily after an initial titration on day 1 until day 5.

Immunotherapy-induced psoriasis arises in the majority of cases as a flare of disease in patients with a positive personal history. Furthermore, the pathogenesis is not completely understood. Tanaka et al. demonstrated that a key mediator of nivolumab-induced psoriasis could be IL-6, but not TNF-a (16).

IL-6 and transforming growth factor (TGF)-b stimulate the activation of T-helper cell 17 (Th17), which produces IL-17, a key cytokine in suppressing Treg cells and in the pathogenesis of inflammatory skin disorders. The balance between Th17 cells and Treg cells is a key factor in the regulation of autoimmunity and cancer (6).

The management of psoriasiform rash as published by Phillips et al. underlines the main role of topical or systemic corticosteroid therapy for Grade 1, adding narrow bands (NB)-UVB phototherapy for Grade 2 (17). Biological agents, including Apremilast, are indicated for an intolerable Grade 2–3 psoriasis, interrupting if necessary immune checkpoint inhibitors until decrease to Grade 0–1.

The use of Apremilast in checkpoint inhibitors-induced psoriasis is controversial, lacking in evidence, and its use is supported by a small case series. Salopek published a case report of a recurrence of melanoma in a young patient after taking Apremilast for the worsening of psoriasis (18). The author hypothesized that the use of Apremilast resulted in impaired cancer immunosurveillance and led to melanoma recurrence (17, 18).

A few case reports were published in non-small cell lung cancer (NSCLC) setting as well (19–21). Fattore et al. reported a case of psoriasis triggered by nivolumab in a woman with NSCLC. Contrary to our case, the patient had a known personal history of psoriasis. Whereas in the ineffectiveness of topical therapy, the dermatologist started Apremilast with no obvious interference with immunotherapy (19).

Sapalidis et al. presented a case report of a patient with lung adenocarcinoma in second-line treatment with nivolumab who underwent psoriatic arthritis. The patient was treated with Apremilast, interrupting immunotherapy (20). Moreover, Apalla et al. reported one case of a woman with lung adenocarcinoma and nivolumab-induced psoriasis treated effectively with Apremilast. Immunotherapy was continued with good response (21).

An interesting case of psoriatic arthritis induced by anti-PD1 treated with Apremilast in a melanoma patient was recently reported by Nigro et al. Nivolumab has been discontinued, achieving disease stability (22).

Apremilast seems to be effective and safe for the treatment of immunotherapy-induced psoriasis but only through individual clinical cases. More evidence is needed in order to give the clinician a well-structured algorithm to apply in this setting. Some key discussion points should be whether to start Apremilast immediately or after the failure of other approaches, if it is possible to resume immunotherapy, how to manage systemic steroid therapy if needed, and the impact of the long-term antitumor response.

## Conclusion

To the best of our knowledge, this is the first evidence of the use of Apremilast in immunotherapy-induced psoriasis in a melanoma patient. In our case, Pembrolizumab induced a strong immune response against melanoma, as demonstrated not only by the complete clinical response but also by a significant skin immune-related reaction. The use of Apremilast, in this case, was effective in managing psoriasis skin lesions without affecting Pembrolizumab anti-cancer activity. Our experience may suggest that PD-1 and PDE-4 concomitant targeting are safe and effective in the immune-related psoriasis management of melanoma disease. Therefore, this evidence warrants further investigation.

## Data Availability Statement

The original contributions presented in the study are included in the article/supplementary material, further inquiries can be directed to the VB, barbieri@unicz.it.

## Ethics Statement

Written informed consent for publication of clinical details and/or clinical images was obtained from the patient.

## Author Contributions

MS and VB wrote the manuscript. MS, VB, MD’A, and SD interacted with the patient. PTas and PTag supervised and critically revised the work. All authors read and approved the final manuscript.

## Conflict of Interest

The authors declare that the research was conducted in the absence of any commercial or financial relationships that could be construed as a potential conflict of interest.
